# Supplementary material for: A Humanized Yeast Phenomic Model of Deoxycytidine Kinase to Predict Genetic Buffering of Nucleoside Analog Cytotoxicity
Source: Genes (Basel). 2019 Sep 30;10(10):770. doi: 10.3390/genes10100770 (PMC6826991; doi:10.3390/genes10100770)
Supplement: Supplementary file 1 [file genes-10-00770-s001.zip › Additional_Files/Additional_File7_GOTermHeatmaps.zip!/Additional_File7_GOTermHeatmaps/holo_TFIIH_complex.pdf]

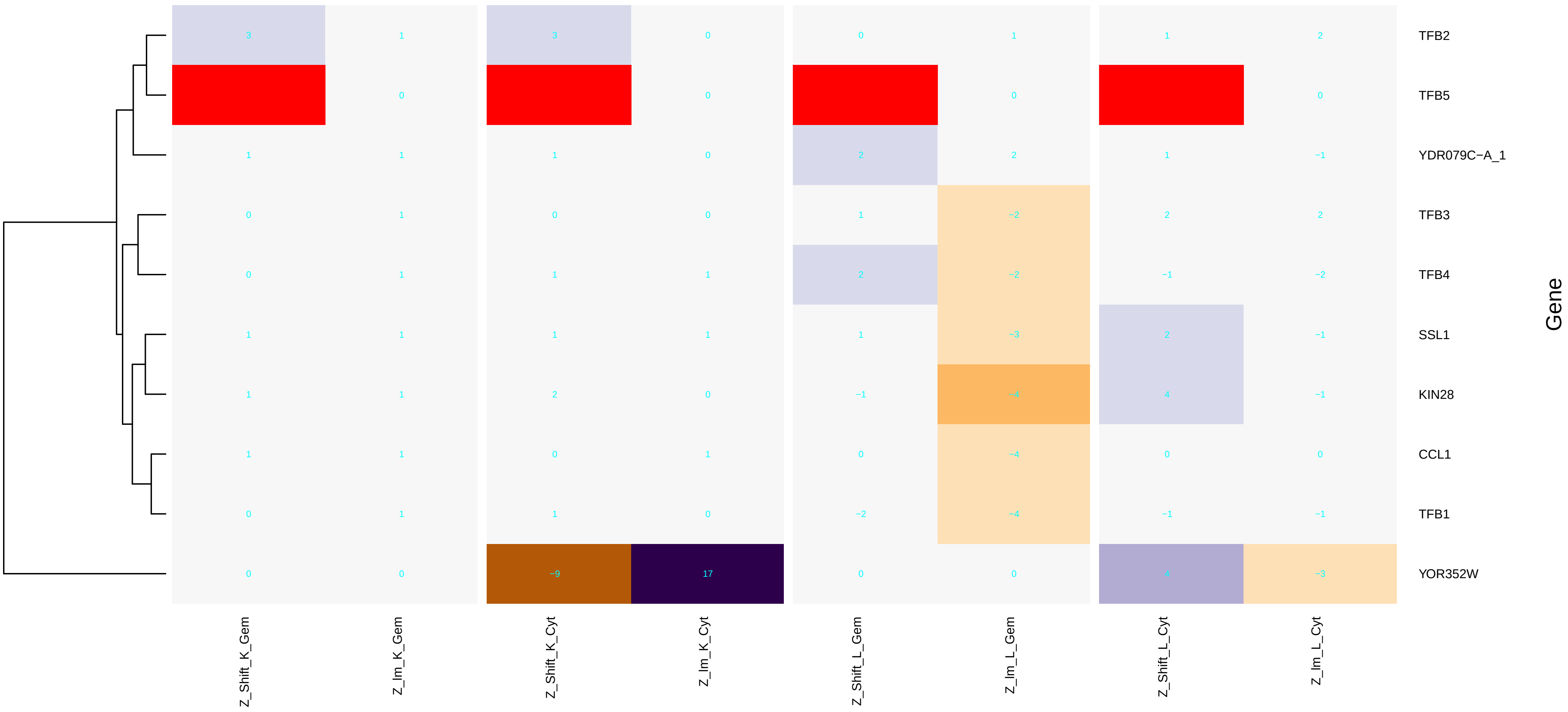

TFB2
TFB5
YDR079C−A_1
TFB3
TFB4
SSL1
KIN28
CCL1
TFB1
YOR352W
Gene
Z_Shift_K_Gem
Z_Im_K_Gem
Z_Shift_K_Cyt
Z_Im_K_Cyt
Z_Shift_L_Gem
Z_Im_L_Gem
Z_Shift_L_Cyt
Z_Im_L_Cyt
3 1 3 0 0 1 1 2
0 0 0 0
1 1 1 0 2 2 1 −1
0 1 0 0 1 −2 2 2
0 1 1 1 2 −2 −1 −2
1 1 1 1 1 −3 2 −1
1 1 2 0 −1 4 −1
1 1 0 1 0 −4 0 0
0 1 1 0 −2 −4 −1 −1
0 0 −9 17 0 0 −3
